# Editorial: Air quality and biosphere-atmosphere interactions

**DOI:** 10.3389/fdata.2025.1611364

**Published:** 2025-05-06

**Authors:** Yves Philippe Rybarczyk

**Affiliations:** School of Information and Engineering, Dalarna University, Falun, Sweden

**Keywords:** air pollutants, machine learning, human health, environmental habitat, human activity

The intricate relationship between *Air quality and biosphere-atmosphere interactions* (BAI) has garnered significant attention in the face of escalating environmental Research Topic. As we navigate the complexities of climate change, understanding how these two domains influence each other becomes paramount not only for elucidating ecological dynamics but also for enhancing human health outcomes. This Research Topic is based on four significant articles bringing a new contribution to both scientific knowledge and policy considerations regarding air quality and environmental health.

The interplay between air quality and BAI is marked by mutual influences; air quality can modulate biospheric processes, while biospheric changes can, in turn, impact air quality. As highlighted by the available literature, the formation of particulate matter (PM) is a critical focal point. PM influences cloud formation and affects regional climate, creating feedback loops that complicate our understanding of ecological systems. The studies presented here delve into various aspects of these interactions, employing cutting-edge methodologies, including machine learning approaches, to elucidate the dynamics at play.

The first article addresses the Rybarczyk et al.. This research underscores the significant correlation between PM_2.5_ concentrations and urban temperatures. Utilizing convergent cross-mapping (CCM), the authors reveal that while urban heat islands (UHIs) exacerbate air pollution levels, high pollution also contributes to elevated urban temperatures. The establishment of a non-linear threshold effect provides a compelling argument for urban planning strategies to address UHI phenomena, particularly in rapidly urbanizing regions like Quito, Ecuador.

The second manuscript focuses on Rodrigues et al.. This innovative study introduces a novel dataset, Trad-204, to assess the susceptibility of Tradescantia plants to environmental stressors caused by air and soil pollution. Using computer vision models to quantify color changes in plant cells, the authors emphasize the potential for deploying biological indicators as a rapid assessment tool for air quality monitoring. This research not only illustrates the sensitivity of biotic systems to pollutants but also introduces advanced neural network architectures that enhance classification accuracy compared to traditional methods.

The third article is a study on the Chaves et al.. Utilizing Random Forest and Long Short-Term Memory (LSTM) neural networks, this work examines PM_2.5_ dynamics in the context of vehicular emissions and meteorological factors. A key finding is the significant impact of the COVID-19 pandemic on vehicle circulation patterns and, consequently, on PM levels. The high predictive accuracy achieved underscores the potential for these models to inform public health policies and strategies for managing urban air quality.

Finally, the last article analyses the Azevedo et al.. This study provides an extensive evaluation of how meteorological variables affect particulate matter levels. By establishing a robust dataset encompassing vehicle counts and PM data, the authors illustrate the interconnected roles of emissions and atmospheric conditions in shaping air quality. Their findings may assist in formulating strategies that emphasize compliance with emissions standards and enhance vehicular management, thus improving urban air quality.

The collective findings of these studies highlight the urgent need for interdisciplinary approaches to tackling air quality Research Topic, integrating knowledge from ecological sciences, public health, and machine learning technologies. As the interactions between air quality and BAI grow more intricate due to climate change and urbanization, future research must focus on refining predictive models and leveraging new technologies.

Exploration of machine learning techniques continues to hold promise, with an imperative to improve the interpretability of models, particularly concerning non-linear dynamics in environmental systems. Addressing current gaps in understanding how anthropogenic activities alter BAI processes will be crucial, particularly as policymakers seek actionable strategies to mitigate climate impacts and protect public health.

In sum, this Research Topic not only advances our scientific understanding of air quality and BAI interactions but also serves as a critical resource for future research trajectories. It is hoped that these contributions will foster heightened awareness and inspire further inquiry into the vital connections that influence both the biosphere and the wellbeing of humanity.

As we continue to face the pressing challenges of environmental degradation, the insights garnered from this body of work serve as a foundation for actionable strategies that bridge ecological resilience and human health in our rapidly changing world.

